# Myristate induces mitochondrial fragmentation and cardiomyocyte hypertrophy through mitochondrial E3 ubiquitin ligase MUL1

**DOI:** 10.3389/fcell.2023.1072315

**Published:** 2023-03-27

**Authors:** César Vásquez-Trincado, Mario Navarro-Márquez, Pablo E. Morales, Francisco Westermeier, Mario Chiong, Valentina Parra, Alejandra Espinosa, Sergio Lavandero

**Affiliations:** ^1^ Facultad de Ciencias Químicas y Farmacéuticas y Facultad de Medicina, Advanced Center for Chronic Diseases (ACCDiS), Universidad de Chile, Santiago, Chile; ^2^ Escuela de Química y Farmacia, Facultad de Medicina, Universidad Andres Bello, Santiago, Chile; ^3^ Departamento de Tecnología Médica, Facultad de Medicina, Universidad de Chile, Santiago, Chile; ^4^ Corporación Centro de Estudios Científicos de las Enfermedades Crónicas (CECEC), Santiago, Chile; ^5^ Division of Cardiology, Department of Internal Medicine, University of Texas Southwestern Medical Center, Dallas, TX, United States

**Keywords:** lipotoxicity, heart, hypertrophy, mitochondria, MUL1, MAPL, insulin-desensitization

## Abstract

**Introduction:** Cardiovascular diseases, especially metabolic-related disorders, are progressively growing worldwide due to high-fat-containing foods, which promote a deleterious response at the cellular level, termed lipotoxicity, or lipotoxic stress. At the cardiac level, saturated fatty acids have been directly associated with cardiomyocyte lipotoxicity through various pathological mechanisms involving mitochondrial dysfunction, oxidative stress, and ceramide production, among others. However, integrative regulators connecting saturated fatty acid-derived lipotoxic stress to mitochondrial and cardiomyocyte dysfunction remain elusive.

**Methods:** Here, we worked with a cardiomyocyte lipotoxicity model, which uses the saturated fatty acid myristate, which promotes cardiomyocyte hypertrophy and insulin desensitization.

**Results:** Using this model, we detected an increase in the mitochondrial E3 ubiquitin ligase, MUL1, a mitochondrial protein involved in the regulation of growth factor signaling, cell death, and, notably, mitochondrial dynamics. In this context, myristate increased MUL1 levels and induced mitochondrial fragmentation, associated with the decrease of the mitochondrial fusion protein MFN2, and with the increase of the mitochondrial fission protein DRP1, two targets of MUL1. Silencing of MUL1 prevented myristate-induced mitochondrial fragmentation and cardiomyocyte hypertrophy.

**Discussion:** These data establish a novel connection between cardiomyocytes and lipotoxic stress, characterized by hypertrophy and fragmentation of the mitochondrial network, and an increase of the mitochondrial E3 ubiquitin ligase MUL1.

## 1 Introduction

Cardiovascular diseases (CVD) are the leading cause of death worldwide and are directly linked with unhealthy behaviors, such as smoking, sedentarism, and an unhealthy diet (World Health Organization, 2014). A high-fat diet (HFD) is among the main pathological drivers of obesity, metabolic syndrome, and insulin resistance, which frequently are underlying diseases of CVD (Ritchie and Dale Abel, 2020). Saturated fatty acids (SFAs) are often associated with a deleterious response termed lipotoxic stress or lipotoxicity, which induces several detrimental effects, such as mitochondrial dysfunction, sphingolipid production (ceramide synthesis), insulin-desensitization, and ultimately, cell death (Choi et al., 2021; Yoon et al., 2021). In this context, saturated long-chain fatty acids, such as myristic and palmitic acid, are usually associated with these pathological processes.

Mitochondrial dynamics, a concept comprising mitochondrial fusion, fission, biogenesis, and mitophagy, determine mitochondrial morphology, quality, abundance, and function (Vásquez-Trincado et al., 2016; Eisner et al., 2018; Giacomello et al., 2020). Regarding mitochondrial fusion and fission balance, mitofusins (MFN) 1/2 and optic atrophy 1 (OPA1) regulate mitochondrial fusion and the GTPase dynamin-related protein 1 (DRP1), mitochondrial fission factor (MFF), adaptor mitochondrial dynamics proteins of 49 and 51 kDa (MID49/51), and mitochondrial fission one protein (FIS1) promote mitochondrial fission. We have previously studied the effect of saturated fatty acids (Kuzmicic et al., 2014) and sphingolipids (Parra et al., 2008) on cardiomyocyte mitochondrial fusion and fission balance, in which these lipidic species promoted a marked mitochondrial fragmentation associated with mitochondrial dysfunction and apoptotic cell death.

The mitochondrial E3 ubiquitin ligase MUL1 (also known as MULAN/MAPL/GIDE) (Li et al., 2008; Zhang et al., 2008; Braschi et al., 2009) is a mitochondria-anchored protein that has been associated with NF-κB signaling, mitochondrial dynamics, cell death, inflammation, AKT regulation, and mitophagy (Li et al., 2008; Braschi et al., 2009; Jung et al., 2011; Bae et al., 2012; Jenkins et al., 2013; Yun et al., 2014). Regarding mitochondrial dynamics, MUL1 is associated with the reduction of MFN2 levels, through ubiquitination-mediated degradation (Puri et al., 2019). Additionally, MUL1 stabilizes DRP1 through SUMOylation, promoting mitochondrial fission (Braschi et al., 2009). MUL1 is also a negative regulator of AKT, a well-known regulator of insulin signaling, and reduces total levels of AKT through ubiquitination (Bae et al., 2012). Considering this background, we hypothesized that MUL1 mediates the detrimental effects of SFAs in cardiomyocytes. To test this, cultured cardiomyocytes were treated with the SFA myristate (14:0). This fatty acid increases cardiomyocyte cell area (Russo et al., 2012). Moreover, myristate has been associated with insulin resistance and metabolic syndrome in humans (18). Interestingly, a milk-fat based diet, with a high content of myristate and myristate-derived sphingolipids, induces profound hyperglycemia and insulin resistance (Russo et al., 2012). Additionally, this diet generates left ventricle (LV) hypertrophy and a reduction of the cardiac ejection fraction, faster than the commonly used HFD (Russo et al., 2012). Thus, myristate closely recapitulates the effects of fatty acid overload at the cardiac level in terms of morphological (hypertrophy) and functional changes (insulin-desensitization). Here, we found that myristate increased MUL1 protein levels in cardiomyocytes, and importantly, MUL1 was required for myristate-induced cardiomyocyte hypertrophy and mitochondrial fragmentation. Interestingly, MUL1 is dispensable for myristate-induced insulin-desensitization in the cardiomyocyte.

## 2 Materials and methods

### 2.1 Isolation of neonatal rat ventricular myocytes (NRVM)

Rats were bred at the Faculty of Chemical and Pharmaceutical Sciences Animal Breeding Facility (University of Chile). NRVM were isolated from the hearts of neonatal Sprague-Dawley rats as previously described (Kuzmicic et al., 2014). Neonatal pups, between 2–3 days old, were decapitated, and the hearts were extracted and washed with Hank’s buffer (Sigma, H2387) supplemented with NaHCO_3_ and HEPES, at 37°C. Cardiac atria were carefully removed, and the remanent ventricular portion was minced. The tissue was then enzymatically digested with type II collagenase (Gibco, 17101–015) (0.02 g/100 mL Hank’s) and pancreatin (Sigma, P3292) (0.06 g/100 mL Hank’s). The cell suspension was pre-plated in a 250 mL culture flask for 2–3 h in DME- (Sigma, D1152) and M199- (Sigma, M2520) containing medium, supplemented with 10% FBS (Biological Industries, 04-121-1A), to obtain a cardiomyocyte-enriched fraction. Cells in suspension were collected and centrifuged at 1,000 r.p.m. for 5 min and then resuspended in 25 mL of DME:M199 (4:1) medium, supplemented with 5% FBS and 10% NBCS (Gibco, 16010–159), to be finally seeded in gelatin 2% p/v (Winkler, GE0820) -coated plates.

All procedures and experiments in animals were performed according to NIH Guide for the Care and Use of Laboratory Animals and approved by the corresponding Institutional Ethics Committee.

### 2.2 Cell culture

NRVM were maintained in DME:M199 (4:1), supplemented with 5% FBS and 10% FCS, in the presence of 5-bromo-2′-deoxyuridine (SAFC, B5002). Before any experimental procedure, NRVM were carefully washed with PBS at 37 °C, and then maintained in DME:M199 (4:1) for 24 h. To prepare the myristate solution conjugated with fatty-acid-free bovine serum albumin (BSA) (Sigma, 126575), BSA was first dissolved in DME:M199 (4:1) to get a final concentration of 100 μM. Sodium myristate (Sigma, M8005) was slowly added to this solution, which was mixed and heated between 40°C–50 °C. To ensure the conjugation of the fatty acid to BSA, the solution was then constantly shaken at 37 °C for 1 h. Using this protocol, we prepared working solutions of 100, 250, and 500 μM of myristate, all of which were conjugated with 100 μM BSA. For all the experiments, 100 μM BSA was used as an experimental control condition. NRVM were incubated for 24 h with myristate or BSA solutions.

In the insulin stimulation experiments, after 24 h of myristate or BSA incubation, NRVM were treated with 10 nM insulin (Actrapid HM, Novo Nordisk) for 15 min or 3 h. The shorter time (15 min) was used to assess the activation of primary insulin receptor signaling components and glucose transport (Gutiérrez et al., 2014). The longer time (3 h) was used to evaluate the metabolic response of mitochondria to insulin (Parra et al., 2014).

### 2.3 NRVM transfection

Small interfering RNAs (siRNAs) for Mul1 (SASI_Rn02_00221478; SASI_Rn02_00221479, Sigma Aldrich) and control siRNA (MISSION siRNA universal negative control, Sigma Aldrich) were prepared and used as indicated by the manufacturer’s guidelines. NRVM were transfected following general guidelines described in (Gutiérrez et al., 2014). Transfection media was OPTI-MEM (Life Technologies, 31985062), using Oligofectamine (Sigma, 12252–011) as a transfection vehicle. The Mul1 siRNA sequences were the following: siRNA Mul1 (1): sense sequence (5′-GGG​AAA​GUG​UGU​GCC​UUA​U-3′); antisense sequence (3′-AUA​AGG​CAC​ACA​CUU​UCC​C-5′) and siRNA Mul1 (2): sense sequence (5′-CUG​AGC​AAC​UUC​AAG​UCU​U-3′); antisense sequence (3′-AAG​ACU​UGA​AGU​UGC​UCA​G-5′).

### 2.4 Cell viability

NVRM were seeded in 24-well plates and then subjected to the corresponding experimental conditions. Loss of viability was assessed by incubating the cells with 1 μg/mL propidium iodide (PI, Sigma, P4170-100 MG) under non-permeabilizing conditions, as similarly described in (Pennanen et al., 2014). Cell fluorescence was examined by flow cytometry (BD Accuri C6).

### 2.5 Cell area determination

NRVM were seeded in 12-well plates with coverslips. After experimental treatments, cells were washed with PBS at 4°C, and then fixed with 4% paraformaldehyde (Electron Microscopy Sciences, 15710) in PBS for 10 min. Cells were then incubated with 100 mM glycine (Amresco, 0167) in PBS for 20 min, permeabilized with 0.1% Triton X-100 (Merck, 108643) in PBS for 30 min, and blocked with 3% BSA (Winkler, 0150) for 1 h. Cells were incubated with rhodamine-phalloidin (Life Technologies, R415) 1:500 for 1 h, as described in (Pennanen et al., 2014). Finally, cells were washed with PBS, and mounted with DAKO (Dako, S3023). Cell area of 25–30 NVRM per experimental condition, from 3-4 different experiments, was quantified using ImageJ.

### 2.6 Western blot analysis

NRVM were seeded in 35 mm plates for this experiment. Total protein extracts were analyzed as previously described (Pennanen et al., 2014). Protein content was normalized with a loading control such as β-TUBULIN. Primary and secondary antibodies, and the corresponding dilutions used, are listed in Table 1.

**TABLE 1:** List of antibodies used.

Antibody	Catalog Number	Dilution	Host
β-MHC	VP-M667	1:1000	Mouse
Insulin receptor (IR)	CST-30255	1:1000	Rabbit
Phospho-IR	CST-30245	1:1000	Rabbit
AKT	CST-9272S	1:1000	Rabbit
Phospho-AKT (S473) XP	CST-4060S	1:1000	Rabbit
ERK1/2	CST-9102S	1:1000	Rabbit
Phospho-ERK1/2	CST-4370S	1:1000	Mouse
FOXO1	CST-2880S	1:1000	Rabbit
Phospho-FOXO1/3a (T24/T32)	CST-9464S	1:1000	Rabbit
MUL1	Ab84067	1:500	Rabbit
MFN2	Ab50838	1:1000	Rabbit
DRP1	BD-611738	1:1000	Mouse
OPA1	Ab42364	1:1000	Rabbit
FIS1	ALX-210-1037-0100	1:1000	Rabbit
mtHSP70	ALX-804-077	1:1000	Mouse
β-TUBULIN	SIGMA T0198	1:5000	Mouse
Anti-Rabbit IgG peroxidase conjugate	EMD 401393	1:5000	Goat
Anti-Mouse IgG peroxidase conjugate	EMD 402335	1:5000	Rabbit

### 2.7 Immunofluorescence assays

NRVM were seeded in 12-well plates with coverslips. Cells were fixed, permeabilized, and blocked, as described above. For FOXO1 detection, cells were then incubated with the anti-FOXO1 primary antibody (Cell signaling Technology, 2880) and the Alexa-Fluo 488 anti-rabbit secondary antibody (Life Technologies, A11008). Compartmentalization analysis of FOXO1 signal was measured from 40–50 NVRM per condition, from 3 different experiments, using ImageJ, as previously described in (Bravo et al., 2011). For mtHSP70 and ceramide detection, NRVM were then incubated with the anti-mtHSP70 (GRP75/MOT) primary antibody (Abcam, 53098) and ceramide antibody (Enzo Life, ALX-804–196-T050) and the Alexa-Fluo 488 and Alexa-Fluo 568 anti-rabbit and anti-mouse secondary antibody, respectively (Life Technologies, A11008, and A11004). Cells were mounted with ProLong Gold (Invitrogen, P36935). Inhibition of ceramide synthase was obtained with 50 nM of Fumonisin B1 (Tocris-Bioscience 3103–1). The number of objects (mitochondria) stained with mtHSP70 immunolabelling was quantified with the ImageJ 3D Object Counter plug-in, following general guidelines provided by Parra et al. (2014).

### 2.8 Mitochondrial dynamics analysis

NRVM were washed with PBS at 37°C and then incubated for 30 min with 400 nM Mitotracker Green (MTG) (ThermoFisher Scientific, M7514) in Krebs buffer. Cells were visualized by confocal microscopy (LSM 700, Carl Zeiss) with a Plan-Apochromat 63X/1.4 Oil DIC objective, after exciting at 488 nm with an argon laser. Images were captured as sequential planes alongside the *Z*-axis, every 0.4–0.5 μm. The images were deconvolved using a Landweber function and the corresponding Point Spread Function (PSF). The number and the individual volume of objects (mitochondria) stained with the MTG probe were quantified with the ImageJ 3D Object Counter plug-in, as was performed in (Parra et al., 2014).

### 2.9 Fluorescence recovery after photobleaching (FRAP)

NRVM were washed with PBS at 37 °C and then incubated for 30 min with 200 nM Tetramethylrhodamine, Methyl Ester, Perchlorate (TMRM) (Invitrogen, T668) in Krebs buffer. TMRM was excited at 561 nm, and fluorescence emission was detected with a 650/710 emission filter. Bleaching of TMRM fluorescence was applied in a ≈20 μm^2^ square, at perinuclear and radial regions. Fluorescence intensity was normalized to the intensity levels before and after bleaching. The images were collected every 0.4–2.0 s and analyzed frame by frame with ImageJ software. Data were analyzed from 15 cells examined in three separate experiments.

### 2.10 Immunoprecipitation assay

Immunoprecipitation of DRP1 and MFN2 was performed overnight using 2 μg of anti-DRP1 antibody (BD-611113) and 2 μg of anti-MFN2 antibody (Ab50838) on 500–800 μg of total protein. DRP1 and MFN2 were precipitated with Sepharose beads conjugated to protein G (Protein A/G PLUS-Agarose, Santa Cruz, SC-2003) resolved by SDS-PAGE. SUMOylation was then assessed with an anti-SUMO1 antibody (Santa Cruz SC-9060) and ubiquitination was determined with anti-K48-linkage specific polyubiquitin antibody (CST-8081). As an experimental control to detect poly-ubiquitination, we used the proteosome inhibitor MG-132 (Calbiochem 474790).

### 2.11 ATP measurement

NRVM were seeded in 24-well plates. After the experimental treatments, cells were washed with Krebs buffer and treated with 50 μL of Cell Titer-Glo lysis buffer (Promega, G7571). The plate was then vigorously shaken for 2 min. The suspension was transferred into a 96-well plate, and the luminescence was measured with a Glomax Multidetection System (Promega), as (Pennanen et al., 2014). The cells were incubated with 1 μM oligomycin (Alomone, O500) for 1 h as a negative control.

### 2.12 Flow cytometry analysis of mitochondrial membrane potential and mitochondrial mass

NRVM were seeded in 24-well plates and incubated with 200 mM TMRM (ThermoFisher Scientific, T668) or 400 nM MTG for 30 min to measure mitochondrial membrane potential or mitochondrial mass, respectively, following general guidelines described in (Parra et al., 2014). Cells were then incubated with 300 μL of 1x Trypsin-EDTA (Biological Industries, 03-051-5B) for 5 min. To stop the enzymatic reaction, 50 μL of FBS was added. Cell fluorescence was examined by flow cytometry (BD Accuri C6). CCCP (Sigma, C2759-1G) (50 μM) and oligomycin (1 μM) were used as negative and positive controls, respectively, for mitochondrial membrane potential determination.

### 2.13 ^3^H-2-deoxiglucose uptake

NRVM were seeded in 12-well plates and glucose uptake was performed as described in (Contreras-Ferrat et al., 2010). After the experimental treatments, cells were washed with HEPES buffer and incubated for 5 min with a Transport Solution containing 10 μM 2-deoxy-D-glucose (Sigma, D6134) and 1.0 μCi/mL ^3^H-2-deoxy-D-glucose (Perkin Elmer, NET328A25UC) in HEPES buffer at room temperature. The stop solution (0.95% m/v NaCl y 20 mM de D-glucose) was immediately added at 4°C and the cells were washed 2–3 times with this solution. Cells were then frozen at −20°C for 1 h. Finally, cells were lysed with 500 μL of NaOH 1N per well, and 350 μL of the total lysate was measured with a scintillation counter (Beckman, LS-6000TA). The remaining volume was used to measure the protein concentration with a BCA assay (ThermoFisher Scientific, 23227).

### 2.14 FOXO1 transcription factor binding sites analysis

Nucleotide sequences from −3000 to +1 bp, around to *Mul1* transcription start sites (TSS) were extracted from NIH Gene database. Sequences were obtained from rat (*Rattus norvegicus*), mouse (*Mus musculus*) and human (*Homo sapiens*). These sequences were analyzed with the JASPAR database (http://jaspar.genereg.net) using the matrix profile for FOXO1 (MA0480.1), which contains FOXOs consensus binding sequence (5′-TTGTTTAC-3′) (Eijkelenboom and Burgering, 2013; Webb et al., 2016), using a threshold (relative profile threshold score) of 85%. This threshold value establishes searching sequences whose score is greater than or equal to 85% of the best possible score for the motif analyzed. The results were expressed as relative score. Relative score is calculated as (W—min)/(max—min), where W is the score of the sequence given the PWM (Position Weight Matrix), min (max) is the minimal (maximal) score that can be obtained from the PWM.

### 2.15 Statistical analysis

Experimental data are expressed as the mean ± SEM of independent experiments. Data were analyzed with GraphPad Prism 9.4.1, using one- or two-way ANOVA or Student’s t-test, and comparisons between groups were performed with the corresponding post-test. Statistical significance was defined as *p* < 0.05.

## 3 Results

### 3.1 Myristate induces cardiomyocyte hypertrophy and insulin-desensitization

**FIGURE 1:**
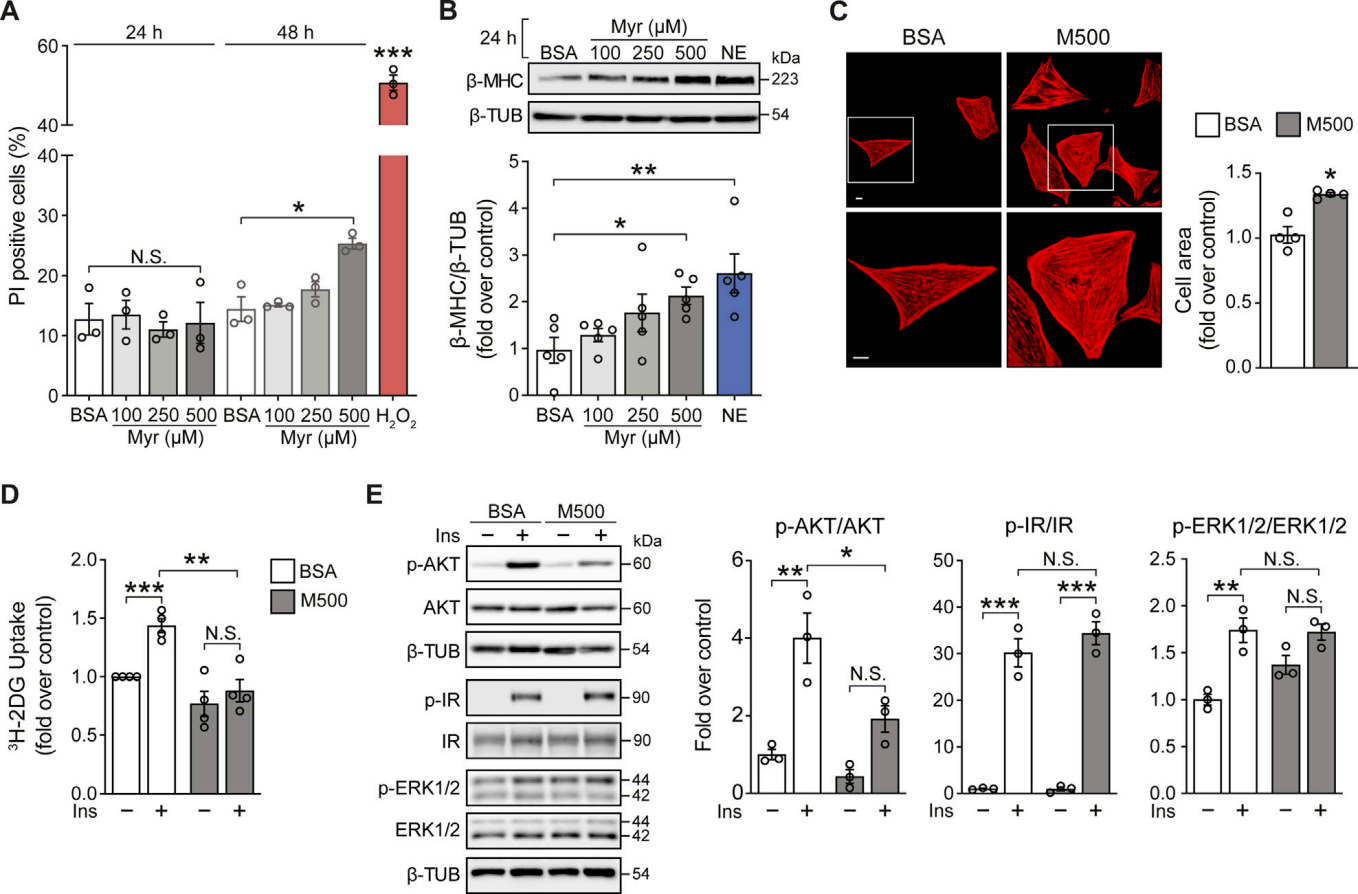
Myristate induces cardiomyocyte hypertrophy and insulin desensitization. **(A)** NRVM were treated with BSA 100 μM or Myristate (Myr) 100, 250 and 500 μM, for 24 and 48 h. Total cell death was evaluated with propidium iodide (PI) incorporation under non-permeabilizing conditions by flow cytometry (n = 3). H_2_O_2_ 500 μM was used as a positive control for cell death. **(B)** *Top*: Representative immunoblot of β-MHC, detected in NRVM treated with Myr 100, 250 and 500 μM, for 24 h. β-TUBULIN (β-TUB) was used as a loading control. Treatment with norepinephrine (NE) 10 μM for 24 h, was used as a positive control for cardiomyocyte hypertrophy. *Bottom*: Quantification of β-MHC/β-TUB (n = 5). **(C)** *Top*: Representative images of NRVM treated with BSA and myristate 500 μM (M500) for 24 h and stained with rhodamine-phalloidin to evaluate cell area and Hoeschst nuclear stain. Scale bar: 10 μm. *Bottom*: Quantification of cell area, from ∼25 NRVM for each experimental condition (n = 4). **(D)** ^3^H-2-deoxyglucose (DG) uptake of NRVM incubated with BSA or M500 for 24 h and stimulated with insulin (Ins) 10 nM for 15 min, or non-stimulated (n = 4). **(E)** *Left*: Representative immunoblots of phospho (p)-AKT (Ser473), total AKT, p-IR (Tyr1150/1151), total IR, p-ERK1/2 (Thr202/Tyr204) and total ERK1/2 from NRVM treated with BSA or M500 for 24 h, unstimulated or stimulated with insulin 10 nM for 15 min. β-TUBULIN was used as a loading control. *Right*: Quantification of p-AKT/AKT, p-IR/IR and p-ERK1/2/ERK1/2 (n = 3). In all panels: individual data points are shown, and bars represent mean ± S.E.M. Statistical comparisons: *p < 0.05, **p < 0.01, and ***p < 0.01. N.S: non-significant.

Cultured neonatal rat ventricular myocytes (NRVM) were incubated with the SFA myristate (100, 250, and 500 μM) for 24 h and 48 h to evaluate the effects of this SFA on cell viability. Exposure of NRVM to myristate for 24 h did not induce cell death in all three concentrations tested (Figure 1A). Incubation for 48 h altered cardiomyocyte morphology (data not shown) and caused cell death using 500 μM of the fatty acid (Figure 1A). Based on these findings, we chose the incubation period of 24 h to determine if myristate induced cardiomyocyte hypertrophy. We evaluated the protein levels of the hypertrophic marker β-MHC, which was increased in NRVM exposed to myristate 500 μM (Figure 1B). Consequently, the cardiomyocyte area was significantly elevated using myristate 500 μM for 24 h (Figure 1C). SFAs have been associated with insulin desensitization and, subsequently, glucose transport impairment. Thus, we tested if myristate altered cardiomyocyte response to insulin by evaluating ^3^H-deoxyglucose uptake. We detected that myristate impairs glucose transport in response to insulin stimulation (Figure 1D). Further analysis of insulin signaling components by immunoblot revealed that AKT phosphorylation (Serine 473 residue) was significantly reduced with the myristate 500 μM treatment (Figure 1E), without any change in total AKT levels (Supplementary Figure S1). Finally, myristate treatment did not affect insulin receptor (IR) activation (Figure 1E); however, it increased (although not significantly) the phosphorylation of ERK proteins, without insulin stimulation.

### 3.2 Myristate increases MUL1 protein levels

**FIGURE 2:**
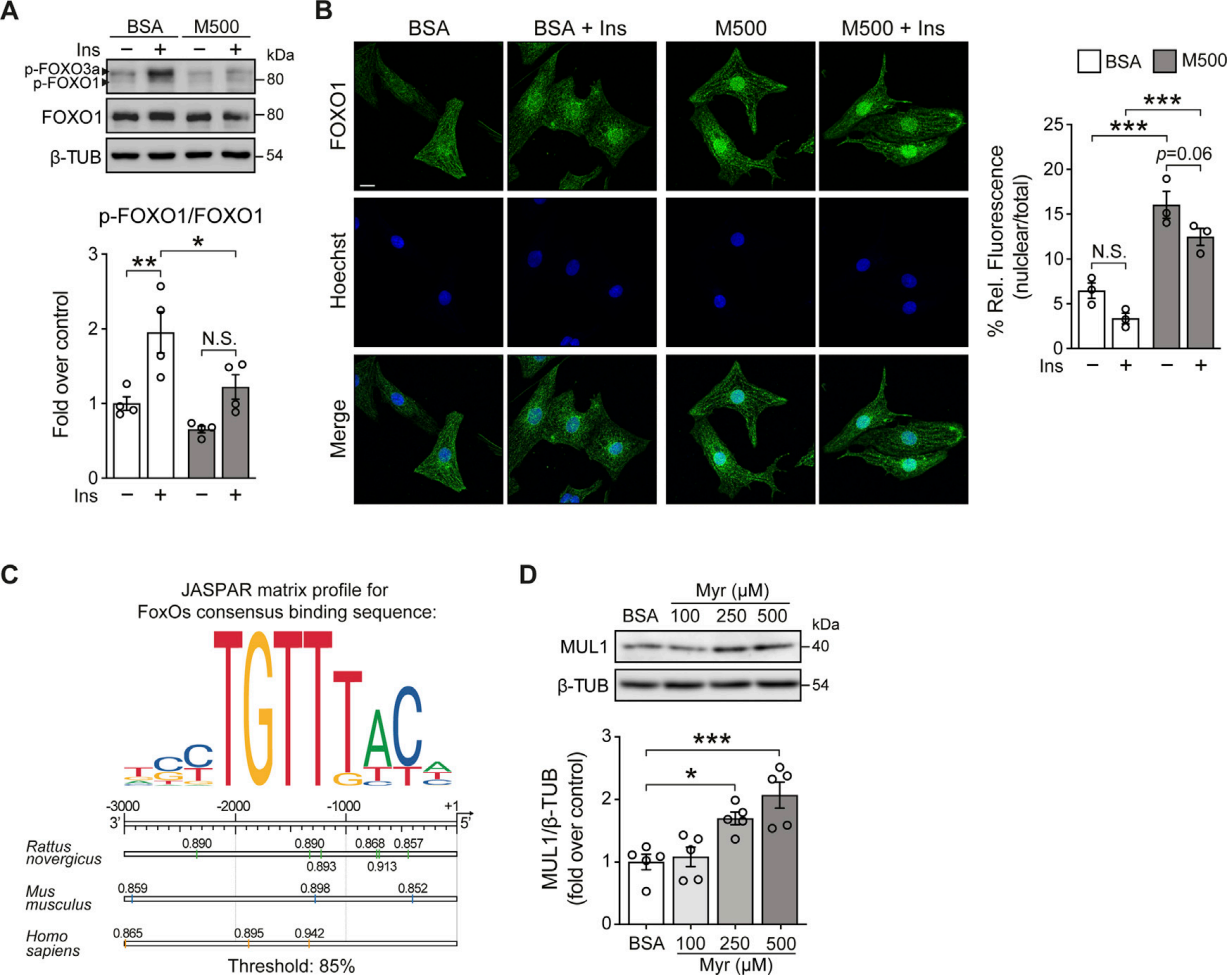
Myristate increases Mul1 protein levels in cultured cardiomyocytes. **(A)** *Top*: Representative immunoblots of phospho (p)-FOXO1/3a (Thr24/Thr32) and total FOXO1 from NRVM treated with BSA or myristate 500 μM (M500) for 24 h, unstimulated or stimulated with insulin 10 nM for 15 min. β-TUBULIN was used as a loading control. *Bottom*: Quantification of p-FOXO1/FOXO1 (n = 4). **(B)** *Left*: Representative immunofluorescence images of NRVM treated with BSA or M500 for 24 h, unstimulated or stimulated with insulin 10 nM for 15 min, using FOXO1 antibody and the Hoechst nuclear stain. Scale bar: 10 μm. *Right*: Quantification of the relative fluorescence of the nuclear compartment (nuclear/total fluorescence), associated with the FOXO1 signal, from 40–50 NRVM per experimental condition (n = 3). **(C)** Top: JASPAR matrix profile used to find FOXO1 binding sites in the *Mul1* promoter region. *Bottom*: Diagram of *Mul1* promoter region from rat, mouse, and human, showing the precise localization of the predicted sequences for FOXO1 binding, with the corresponding relative score, related to the sequence analyzed and matrix utilized. A threshold value of 85% was used. This threshold value establishes searching sequences whose score is greater than or equal to 85% of the best possible score for the motif analyzed. **(D)** *Top*: Representative immunoblot of MUL1, detected in cardiomyocytes treated with Myr 100, 250 and 500 μM, for 24 h. β-TUBULIN (β-TUB) was used as a loading control. *Bottom*: Quantification of MUL1/β-TUB (n = 5). In all panels: individual data points are shown, and bars represent mean ± S.E.M. Statistical comparisons: *p < 0.05, *p < 0.01 and ***p < 0.01.

FOXOs transcription factors are the relevant target of AKT. They are important regulators of cell metabolism and survival (Eijkelenboom and Burgering, 2013). Upon growth factor stimulation, such as insulin, active AKT phosphorylates FOXO, resulting in increased cytosolic localization of FOXO. Conversely, under cellular stress, FOXO translocates to the nucleus and shows increased transcription factor activity (Battiprolu et al., 2012; Eijkelenboom and Burgering, 2013). Since myristate 500 μM impairs insulin signaling in NRVM at AKT level, we evaluated FOXO phosphorylation by immunoblot. Due to its relevance in cardiac metabolism (Battiprolu et al., 2012), we specifically focused on FOXO1. We found that myristate significantly reduced insulin-induced FOXO1 phosphorylation (Threonine 24 residue) (Figure 2A). Evaluation of FOXO1 subcellular localization by immunofluorescence, showed a decrease of FOXO1 nuclear signal, accompanied by enrichment at the perinuclear portion, with insulin stimulation, in NRVM incubated with BSA (Figure 2B). In contrast, we detected, with the exposure to myristate 500 μM, an increase in the nuclear localization, therefore increased transcriptional activity, of FOXO1 in NRVM, which was persistent upon insulin action (Figure 2B). One protein that has been associated with FOXO1/O3 transcriptional activity is the mitochondrial E3 ubiquitin ligase MUL1, a novel regulator of mitochondrial dynamics and several other cellular functions (Calle et al., 2022). We analyzed the promoter region of the *Mul1* gene (from human, rat and mouse) and found several binding sites for FOXO1, using FOXOs consensus binding sequence 5′-TTGTTTAC-3’ (Eijkelenboom and Burgering, 2013) (Figure 2C). Finally, to determine if myristate exposure increased MUL1 protein levels, we stimulated NRVM with myristate (100, 250, and 500 μM) for 24 h, and we found a significant increase of MUL1 levels with myristate 250 and 500 μM (Figure 2D).

### 3.3 Myristate induces mitochondrial fragmentation

**FIGURE 3:**
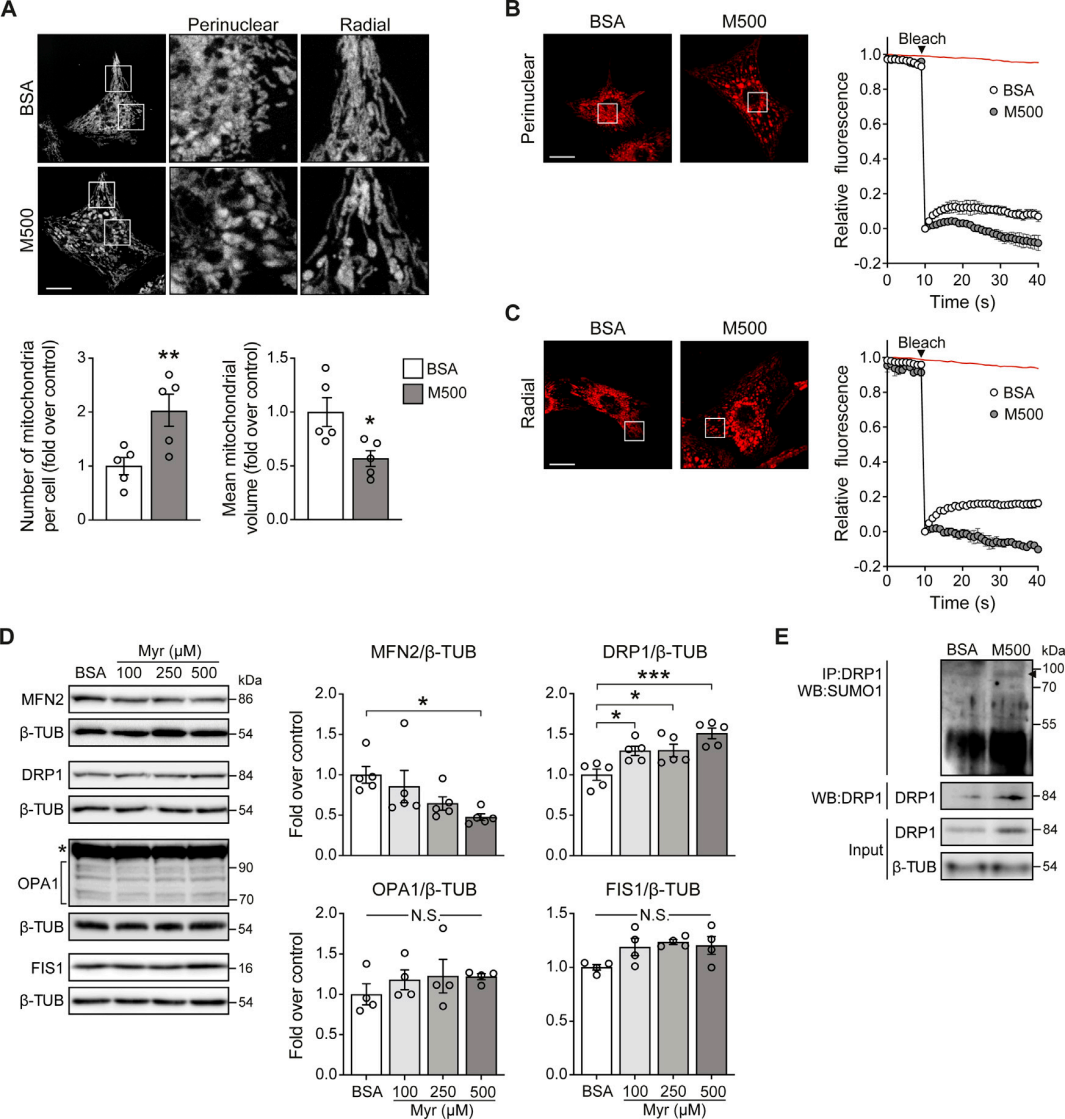
Myristate induces mitochondrial fragmentation in cultured cardiomyocytes. **(A)** *Top*: Representative images of NRVM treated with BSA or myristate 500 μM (M500) for 24 h and stained with MTG to visualize the mitochondrial network. Scale bar = 10 μm. *Bottom*: Quantification of mitochondrial number and average mitochondrial volume (n = 5). **(B)** *Left*: Representative image from FRAP analysis of the perinuclear mitochondrial network of NRVM treated with BSA or M500 for 24 h. Bleaching of TMRM fluorescence was applied in a square at chosen region (perinuclear). *Right*: Quantification of TMRM fluorescence levels. Fluorescence intensity was normalized to the intensity levels before and after bleaching. Red line in the graph indicates the fluorescence decay during the acquisition in a non-bleached area. **(C)** *Left*: Representative image from FRAP analysis of the radial mitochondrial network of NRVM treated with BSA or M500 for 24 h. Bleaching of TMRM fluorescence was applied in a square at chosen region (radial). *Right*: Quantification of TMRM fluorescence levels. **(D)** Representative immunoblots of MFN2, DRP1, OPA1 and FIS1, detected in NRVM treated with Myr 100, 250 and 500 μM, for 24 h β-TUBULIN (β-TUB) was used as a loading control. *Right*: Quantification of MFN2/β-TUB, DRP1/β-TUB, OPA1/β-TUB and FIS1/β-TUB (n = 4–5). **(E)** DRP1 SUMOylation (SUMO1) assessed by immunoblot after immunoprecipitation of DRP1 protein from NRVM treated with BSA or myristate 500 μM (M500) for 24 h. A representative image is shown from three independent experiments with similar outcomes. In all panels: individual data points are shown, and bars represent mean ± S.E.M. Statistical comparisons: *p < 0.05, **p < 0.01, and ***p < 0.01. N.S: non-significant.

MUL1 has been associated with mitochondrial fragmentation through two mechanisms involving reduction of the mitochondrial fusion protein MFN2 and an increment of the mitochondrial fission protein DRP1 (Braschi et al., 2009; Ren et al., 2019). To determine whether myristate induced the fragmentation of the mitochondrial network, NRVM were exposed to myristate 500 μM for 24 h and stained with the mitochondrial-specific dye MitoTracker Green (MTG). Myristate induced disruption of the mitochondrial network connectivity, especially in the perinuclear region (Figure 3A, top). Quantification of the number and volume of individual mitochondria showed that myristate increased the number of mitochondria per cell (Figure 3A, bottom) and, concomitantly, decreased mitochondrial volume (Figure 3A, bottom). To functionally address mitochondrial connectivity, we performed FRAP experiments, which showed a markedly halted fluorescence recovery in myristate-treated NRVM, at both perinuclear (Figure 3B) and radial (Figure 3C) regions. Lipotoxicity is usually associated with ceramides, a class of sphingolipids involved in cardiovascular diseases (Choi et al., 2021). Inhibition of ceramide production has been shown to have beneficial effects at the cardiomyocyte level, especially under myristate exposition (Russo et al., 2012). To test if mitochondrial network fragmentation induced by myristate 500 μM, is associated with ceramide production, we used Fumonisin B_1_ (FB_1_), an inhibitor of ceramide synthases (Riley and Merrill Jr 2019). We obtained that M500-dependent mitochondrial network fragmentation, denoted by the increase in the number of mitochondria per cell, is partially inhibited with FB_1_ (Supplemental Figure S3A).

To address the specific mechanisms behind myristate-induced mitochondrial fragmentation, we measured the protein levels of mitochondrial dynamics components, namely, MFN2, OPA1 (mitochondrial fusion), DRP1, and FIS1 (mitochondrial fission). Cells exposed to myristate 100, 250, and 500 μM for 24 h exhibited a decrease in MFN2 protein levels, specifically with myristate 500 μM (Figure 3D). On the other hand, all three concentrations of myristate increased DRP1 protein levels (Figure 3D). Neither OPA1 nor FIS1 protein levels were changed by myristate treatment (Figure 3D). Because MUL1 induces MFN2 reduction through ubiquitination and DRP1 stabilization through SUMOylation, we tested if myristate 500 μM exposition for 24 h, increases these specific post-translational modifications on MFN2 and DRP1. NRVM incubated with myristate, showed an increase in DRP1-SUMOylation (Figure 3E). We were unable to detect MFN2 ubiquitination in either BSA- or myristate-treated NRVM (Supplemental Figure S3B). However, a detectable signal was visible in NRVM exposed to myristate, when proteosome inhibitor MG132 was used (Supplemental Figure S3B). MUL1 has also been associated with the regulation of mitochondrial clearance through mitophagy (Yun et al., 2014). We determined mitochondrial mass in NRVM treated with myristate (100, 250, and 500 μM for 24 h), using MTG and mtHSP70 as a marker of mitochondrial mass. We observed no variations of MTG intensity (Supplemental Figure S3C) nor of mtHSP70 protein levels with all fatty acid concentrations tested (Supplemental Figure S3D). Thus, treatment of NRVM with myristate 500 μM for 24 h induced mitochondrial fragmentation, associated with the decrease of the mitochondrial fusion protein MFN2 and the increase of the mitochondrial fission protein DRP1.

### 3.4 *Mul1* knockdown prevented myristate-induced mitochondrial fragmentation and cardiomyocyte hypertrophy

**FIGURE 4:**
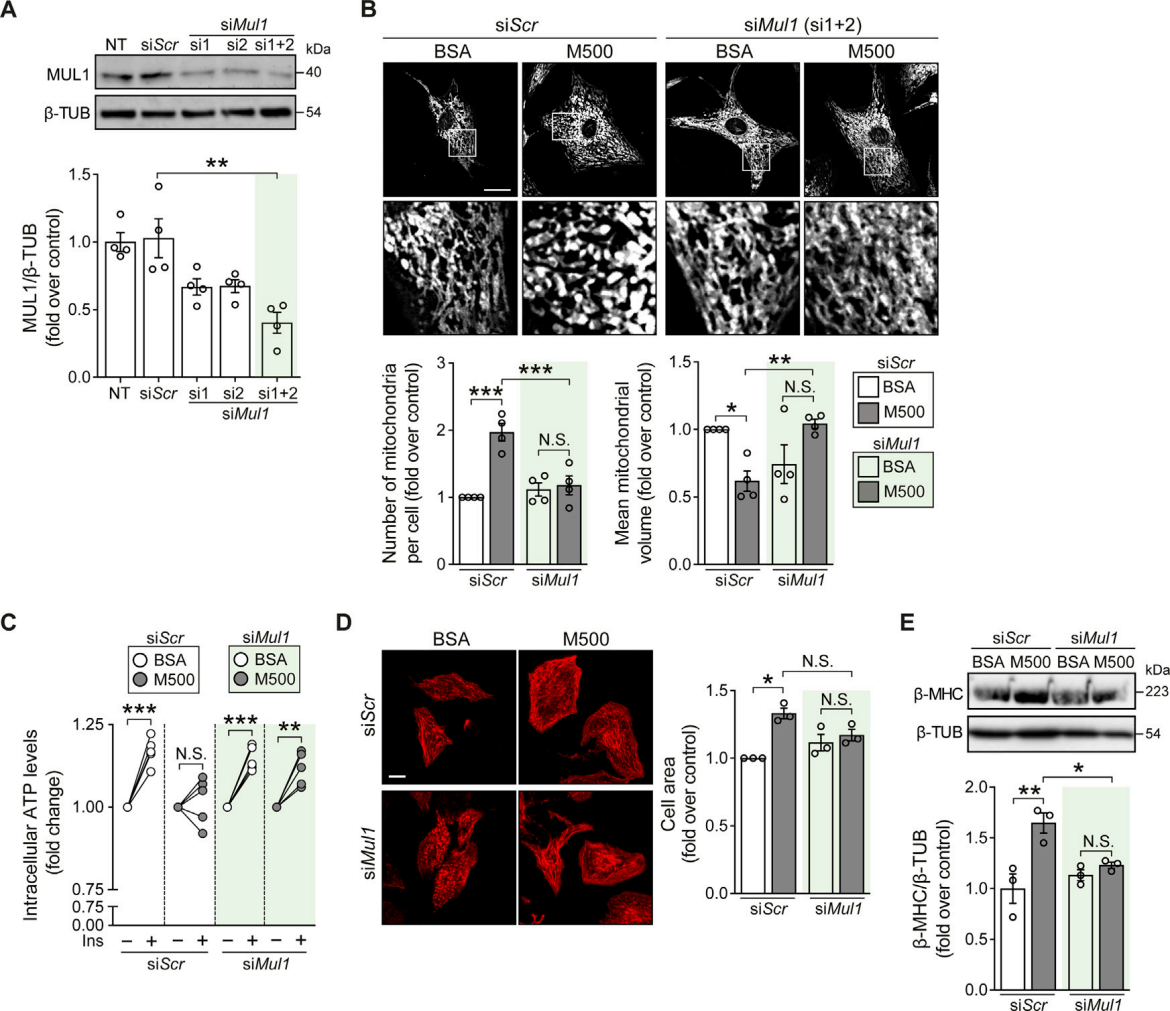
*Mul1*-KD prevents myristate-induced cardiomyocyte hypertrophy and mitochondrial fragmentation. **(A)** *Top*: Representative immunoblot of MUL1, detected in NRVM treated with scrambled siRNA (si*Scr*) or siRNA against *Mul1* (si1 or/and si2). β-TUBULIN (β-TUB) was used as a loading control. *Bottom*: Quantification of MUL1/β-TUB (n = 4). **(B)** *Top*: Representative images of NRVM treated with scrambled siRNA (siScr) or *Mul1* siRNA (si*Mul1*), incubated with BSA or myristate 500 μM (M500) for 24 h and stained with MitoTracker Green to visualize the mitochondrial network. Scale bar = 10 μm. *Bottom*: Quantification of mitochondrial number and average mitochondrial volume (n = 4). **(C)** Measurement of intracellular ATP levels of NRVM treated with scrambled siRNA (siScr) or *Mul1* siRNA (si*Mul1*), incubated with BSA or myristate 500 μM (M500) for 24 h and stimulated with insulin (Ins) 10 nM for 3 h, or non-stimulated (n = 5). **(D)** *Left*: Representative images of NRVM treated with scrambled siRNA (siScr) or Mul1 siRNA (*siMul1*), incubated with BSA and myristate 500 μM (M500) for 24 h and stained with rhodamine-phalloidin to evaluate cell area. *Right*: Quantification of cell area (n = 3). **(E)** *Top*: Representative immunoblot of β-MHC, detected in NRVM treated with scrambled siRNA (siScr) or *Mul1* siRNA (si*Mul1*), incubated with BSA or myristate 500 μM (M500) for 24 h β-TUBULIN (β-TUB) was used as a loading control. *Bottom*: Quantification of β-MHC/β-TUB (n = 3). In all panels: individual data points are shown, and bars represent mean ± S.E.M. Statistical comparisons: *p < 0.05, **p < 0.01, and ***p < 0.01. N.S: non-significant.

Finally, we tested if *Mul1* knockdown (KD) could prevent the lipotoxic effects of myristate on NRVM. Using two oligonucleotides against the *Mul1* mRNA sequence, we obtained a significant decrease in MUL1 protein levels (Figure 4A). Next, we evaluated whether Mul1-KD prevented myristate-induced mitochondrial fragmentation. We found that preincubation with the siRNA against *Mul1* prevented myristate-induced mitochondrial fragmentation, as evaluated by the quantification of mitochondrial number and volume (Figure 4B). As a functional evaluation of enhanced mitochondrial interconnectivity, NRVM were stimulated with insulin for 3 h to boost mitochondrial function (Parra et al., 2014). Since NRVM ATP production is strongly supported by mitochondrial oxidative phosphorylation (Supplemental Figure S4A), we measured intracellular ATP levels as a surrogate of mitochondrial ATP production. Treatment with insulin for 3 h increased intracellular ATP levels, which was halted after incubating the cells with myristate 500 μM for 24 h (Figure 4C). Knockdown of *Mul1* reestablished insulin-induced ATP increase (Figure 4C) and additionally, restored mitochondrial membrane potential response elicited by insulin (Supplemental Figure S4B). Mitochondrial fission has been associated with cardiomyocyte hypertrophy (Pennanen et al., 2014), so we next evaluated the participation of MUL1 in this morphological change induced by myristate. We observed no change in cardiomyocyte cell area upon stimulation with myristate 500 μM (Figure 4D) and, consequently, no increase in the hypertrophic marker β-MHC after silencing *Mul1* (Figure 4E). Finally, we evaluated the effects of *Mul1*-KD on insulin-dependent glucose uptake. The reduction of MUL1 levels did not improve insulin-stimulated glucose uptake altered by myristate incubation (Supplemental Figure S4C). Similarly, in myristate-treated NRVM, activation of AKT upon insulin stimulation exhibited no improvement in the presence of the *Mul1* siRNA (Supplemental Figure S4D).

## 4 Discussion

Our findings showed that lipotoxic stress due to the exposure of NRVM to the SFA myristate, increased the protein levels of the mitochondrial E3 ubiquitin ligase MUL1. To our knowledge, this is the first evidence linking lipotoxicity and MUL1 at the cardiac level. We found that MUL1 is required for myristate-induced cardiomyocyte hypertrophy and mitochondrial fragmentation. A previous study linked MUL1 to phenylephrine-induced cardiomyocyte hypertrophy and mitochondrial fission by decreasing MFN2 (Zhao et al., 2017). In our *in vitro* lipotoxicity model, cardiomyocytes exhibited mitochondrial fragmentation associated with reduced MFN2 levels and, additionally, increased DRP1 levels. Both MFN2 and DRP1 have been reported as MUL1 targets, whose levels also concomitantly change in other pathological conditions, such as ischemic stroke (Ren et al., 2019). MUL1 reduces MFN2 levels through ubiquitination and stabilizes DRP1 through SUMOylation. In myristate-exposed NRVM, we only could detect DRP1 SUMOylation. Originally, MUL1 was described to function as efficient SUMO1 E3 ligase, rather than a ubiquitin ligase, at a physiological range of ubiquitin and SUMO substrates (Braschi et al., 2009). Interestingly, DRP1 SUMOylation has been linked to apoptosis (Prudent et al., 2015), however in our model, myristate incubation for 24 h, was not associated with a significant increase in NRVM cell death (Figure 1A). It could be possible that MUL1-dependent DRP1 SUMOylation is a signal which is activated under elevated cellular stress, like lipotoxicity, and could connect mitochondrial fragmentation with cell death, if the stress conditions surpass an intensity or time threshold. The serine/threonine kinase AKT is another reported target of MUL1 (Bae et al., 2012; Kim et al., 2017; Zhao et al., 2020). In our model, we observed no change in total AKT levels with myristate or the siRNA against MUL1. However, it is important to consider that MUL1-dependent AKT degradation involves a complex interplay between the ubiquitin-proteasome system (UPS) and the autophagy-lysosome pathway (Kim et al., 2021). Another possible explanation could be related to the cell type-dependent selectivity of MUL1 for its targets, where different properties of the cell type, such as metabolic rate, differentiation state, functional profile, mitochondrial abundance, and organelle communication, influence the accessibility of MUL1 to its targets.

Myristate-induced cardiomyocyte hypertrophy and insulin desensitization was associated with reduced phosphorylation and increased nuclear localization of FOXO1 (Figure 2B). Particularly, FOXO1 is a known regulator of cardiac remodeling in physiological conditions such as exercise (Weeks et al., 2021) and in pathological conditions such as pressure overload (Ferdous et al., 2020), and specially, metabolic overload (Battiprolu et al., 2012), which is directly connected to insulin-desensitized conditions, where AKT activity is decreased, and therefore, FOXO1 transcriptional activity is elevated (Eijkelenboom and Burgering, 2013). Importantly, deletion of *Foxo1* in the heart, prevented cardiac hypertrophy and steatosis, in HFD-fed mice (Battiprolu et al., 2012), and additionally, *Foxo1* KO blunted TAC (transverse aortic constriction)-induced cardiac hypertrophic growth (Ferdous et al., 2020). In this context, a possible mechanism for MUL1 increase in the cardiomyocytes exposed to a metabolic overload, such as myristate exposition, might be FOXO1-dependent transcription. Due to the presence of several FOXO1-binding sites in the *Mul1* promoter, it is possible that FOXO1 regulates MUL1 levels in the cardiomyocyte. Additionally, it has been reported that MUL1 levels are regulated by the transcription factor FOXO3, another member of FOXO family, in cancer cells (Kim et al., 2017) and primary myotubes (Sanchez et al., 2018).

An important finding of our work is that *Mul1*-KD prevented myristate-induced mitochondrial fragmentation and cardiomyocyte hypertrophy. Our group has previously reported the relationship between the balance of mitochondrial network and cardiomyocyte hypertrophy (Pennanen et al., 2014). Remarkably, MFN2-KD is sufficient to induce mitochondrial fragmentation and cardiomyocyte hypertrophy. Conversely, the inhibition of mitochondrial fission prevents NE-induced mitochondrial dysfunction and cardiomyocyte hypertrophy (Pennanen et al., 2014). Similarly, pharmacological inhibition of DRP1, using mdivi-1, prevents phenylephrine-induced cardiomyocyte hypertrophy (Liu et al., 2020). Therefore, maintaining a fused mitochondrial network represents a beneficial approach to preventing cardiomyocyte hypertrophic growth, as *Mul1*-KD did in the myristate-induced cardiomyocyte hypertrophy. Keeping the mitochondrial network interconnection, might improve the metabolic adaptation and energy production (as ATP) of cardiomyocytes to a metabolic overload (such as SFA exposition) or an anabolic stimulus, such as insulin. Mitochondrial network connectivity allows efficient distribution of the potential energy through the cell, especially in skeletal muscle and heart (Glancy et al., 2017). In this context, silencing of *Mul1* increased mitochondrial network connectivity, improving mitochondrial energy production and membrane potential transmission along the network, under metabolic overload, with myristate, and insulin stimulation. This was evidenced by the evaluation of mitochondrial network (Figure 4B), intracellular ATP production (Figure 4C) and mitochondrial membrane potential (Supplemental Figure S4B). In a similar way, calcineurin deletion in skeletal muscle, enhanced mitochondrial elongation, and consequently, mitochondrial ATP-coupled respiration (Pfluger et al., 2015). This effect was considered protective against a metabolic overload condition, such as diet-induced obesity.

This study established a novel link between cardiomyocyte lipotoxic stress and MUL1 and, given the increasing number of metabolic-induced CVD, is highly relevant and will help identify potential therapeutical targets or explore mitochondrial dynamics-derived therapies in CVD.

## Data Availability

The original contributions presented in the study are included in the article/Supplementary Material, further inquiries can be directed to the corresponding author.

## References

[B1] Bae S. Kim S. Y. Jung J. H. Yoon Y. Cha H. J. Lee H. (2012). Akt is negatively regulated by the MULAN E3 ligase. Cell Res. 22 (5), 873–885. 10.1038/cr.2012.38 22410793 PMC3343661

[B2] Battiprolu P. K. Hojayev B. Jiang N. Wang Z. V. Luo X. Iglewski M. (2012). Metabolic stress - induced activation of FoxO1 triggers diabetic cardiomyopathy in mice. J. Clin. Investigation 122 (3), 1109–1118. 10.1172/JCI60329 PMC3287230 22326951

[B3] Braschi E. Zunino R. McBride H. M. (2009). MAPL is a new mitochondrial SUMO E3 ligase that regulates mitochondrial fission. EMBO Rep. 10 (7), 748–754. 10.1038/embor.2009.86 19407830 PMC2727426

[B4] Bravo R. Vicencio J. M. Parra V. Troncoso R. Munoz J. P. Bui M. (2011). Increased ER-mitochondrial coupling promotes mitochondrial respiration and bioenergetics during early phases of ER stress. J. Cell Sci. 124 (14), 2143–2152. 10.1242/jcs.080762 21628424 PMC3113668

[B5] Calle X. Garrido-Moreno V. Lopez-Gallardo E. Norambuena-Soto I. Martinez D. Penaloza-Otarola A. (2022). Mitochondrial E3 ubiquitin ligase 1 (MUL1) as a novel therapeutic target for diseases associated with mitochondrial dysfunction. IUBMB Life 74 (9), 850–865. 10.1002/iub.2657 35638168

[B6] Choi R. H. Tatum S. M. Symons J. D. Summers S. A. Holland W. L. (2021). Ceramides and other sphingolipids as drivers of cardiovascular disease. Nat. Rev. Cardiol. 18 (10), 701–711. 10.1038/s41569-021-00536-1 33772258 PMC8978615

[B7] Contreras-Ferrat A. E. Toro B. Bravo R. Parra V. Vasquez C. Ibarra C. (2010). An inositol 1,4,5-triphosphate (IP3)-IP3 receptor pathway is required for insulin-stimulated glucose transporter 4 translocation and glucose uptake in cardiomyocytes. Endocrinology 151 (10), 4665–4677. 10.1210/en.2010-0116 20685879

[B8] Eijkelenboom A. Burgering B. M. T. (2013). FOXOs: Signalling integrators for homeostasis maintenance. Nat. Rev. Mol. Cell Biol. 14 (2), 83–97. 10.1038/nrm3507 23325358

[B9] Eisner V. Picard M. Hajnóczky G. (2018). Mitochondrial dynamics in adaptive and maladaptive cellular stress responses. Nat. Cell Biol. 20 (7), 755–765. 10.1038/s41556-018-0133-0 29950571 PMC6716149

[B10] Ferdous A. Wang Z. V. Luo Y. Li D. L. Luo X. Schiattarella G. G. (2020). FoxO1–Dio2 signaling axis governs cardiomyocyte thyroid hormone metabolism and hypertrophic growth. Nat. Commun. 11 (1), 2551. 10.1038/s41467-020-16345-y 32439985 PMC7242347

[B11] Giacomello M. Pyakurel A. Glytsou C. Scorrano L. (2020). The cell biology of mitochondrial membrane dynamics. Nat. Rev. Mol. Cell Biol. 21 (4), 204–224. 10.1038/s41580-020-0210-7 32071438

[B12] Glancy B. Hartnell L. M. Combs C. A. Femnou A. Sun J. Murphy E. (2017). Power grid protection of the muscle mitochondrial reticulum. Cell Rep. 19 (3), 487–496. Available at: https://www.sciencedirect.com/science/article/pii/S2211124717304242 . 10.1016/j.celrep.2017.03.063 28423313 PMC5490369

[B13] Gutiérrez T. Parra V. Troncoso R. Pennanen C. Contreras-Ferrat A. Vasquez-Trincado C. (2014). Alteration in mitochondrial Ca2+ uptake disrupts insulin signaling in hypertrophic cardiomyocytes. Cell Commun. Signal. 12 (1), 68. 10.1186/s12964-014-0068-4 25376904 PMC4234850

[B14] Jenkins K. Khoo J. J. Sadler A. Piganis R. Wang D. Borg N. A. (2013). Mitochondrially localised MUL1 is a novel modulator of antiviral signaling. Immunol. Cell Biol. 91 (4), 321–330. Available at: https://onlinelibrary.wiley.com/doi/abs/10.1038/icb.2013.7 . 23399697 10.1038/icb.2013.7

[B15] Jung J. H. Bae S. Lee J. Y. Woo S. R. Cha H. J. Yoon Y. (2011). E3 ubiquitin ligase Hades negatively regulates the exonuclear function of p53. Cell Death Differ. 18 (12), 1865–1875. 10.1038/cdd.2011.57 21597459 PMC3214910

[B16] Kim H. J. Kim S. Y. Kim D. H. Park J. S. Jeong S. H. Choi Y. W. (2021). Crosstalk between HSPA5 arginylation and sequential ubiquitination leads to AKT degradation through autophagy flux. Autophagy 17 (4), 961–979. 10.1080/15548627.2020.1740529 32164484 PMC8078769

[B17] Kim S.-Y. Jeong Kim H. Kwon Byeon H. Ho Kim D. Kim C.-H. (2017). FOXO3 induces ubiquitylation of AKT through MUL1 regulation. Available at: www.impactjournals.com/oncotarget . 10.18632/oncotarget.22793 PMC5746397 29299162

[B18] Kuzmicic J. Parra V. Verdejo H. E. Lopez-Crisosto C. Chiong M. Garcia L. (2014). Trimetazidine prevents palmitate-induced mitochondrial fission and dysfunction in cultured cardiomyocytes. Biochem. Pharmacol. 91 (3), 323–336. 10.1016/j.bcp.2014.07.022 25091560

[B19] Li W. Bengtson M. H. Ulbrich A. Matsuda A. Reddy V. A. Orth A. (2008). Genome-wide and functional annotation of human E3 ubiquitin ligases identifies MULAN, a mitochondrial E3 that regulates the organelle’s dynamics and signaling. PLoS ONE 3, e1487. Available at: https://dx.plos.org/10.1371/journal.pone.0001487 . 10.1371/journal.pone.0001487 18213395 PMC2198940

[B20] Liu Y. Xia P. Chen J. Bandettini W. P. Kirschner L. S. Stratakis C. A. (2020). PRKAR1A deficiency impedes hypertrophy and reduces heart size. Physiol. Rep. 8 (6), e14405. 10.14814/phy2.14405 32212257 PMC7093752

[B21] Lovejoy J. C. Champagne C. M. Smith S. R. DeLany J. P. Bray G. A. Lefevre M. (2001). Relationship of dietary fat and serum cholesterol ester and phospholipid fatty acids to markers of insulin resistance in men and women with a range of glucose tolerance. Metabolism - Clin. Exp. 50 (1), 86–92. 10.1053/meta.2001.19440 11172480

[B22] Parra V. Eisner V. Chiong M. Criollo A. Moraga F. Garcia A. (2008). Changes in mitochondrial dynamics during ceramide-induced cardiomyocyte early apoptosis. Cardiovasc. Res. 77 (2), 387–397. 10.1093/cvr/cvm029 18006463

[B23] Parra V. Verdejo H. E. Iglewski M. Del Campo A. Troncoso R. Jones D. (2014). Insulin stimulates mitochondrial fusion and function in cardiomyocytes via the Akt-mTOR-NFκB-Opa-1 signaling pathway. Diabetes 63 (1), 75–88. 10.2337/db13-0340 24009260 PMC3868041

[B24] Pennanen C. Parra V. Lopez-Crisosto C. Morales P. E. Del Campo A. Gutierrez T. (2014). Mitochondrial fission is required for cardiomyocyte hypertrophy mediated by a Ca2+-calcineurin signaling pathway. J. Cell Sci. 127 (12), 2659–2671. Available at: http://jcs.biologists.org/content/127/12/2659.abstract . 10.1242/jcs.139394 24777478 PMC4058110

[B25] Pfluger P. T. Kabra D. G. Aichler M. Schriever S. C. Pfuhlmann K. Garcia V. C. (2015). Calcineurin links mitochondrial elongation with energy metabolism. Cell Metab. 22 (5), 838–850. 10.1016/j.cmet.2015.08.022 26411342

[B26] Prudent J. Zunino R. Sugiura A. Mattie S. Shore G. C. McBride H. M. (2015). MAPL SUMOylation of Drp1 stabilizes an ER/mitochondrial platform required for cell death. Mol. Cell 59 (6), 941–955. 10.1016/j.molcel.2015.08.001 26384664

[B27] Puri R. Cheng X. T. Lin M. Y. Huang N. Sheng Z. H. (2019). Mul1 restrains Parkin-mediated mitophagy in mature neurons by maintaining ER-mitochondrial contacts. Nat. Commun. 10 (1), 3645. 10.1038/s41467-019-11636-5 31409786 PMC6692330

[B28] Ren K. Liu W. N. Tian J. Zhang Y. Y. Peng J. J. Zhang D. (2019). Mitochondrial E3 ubiquitin ligase 1 promotes brain injury by disturbing mitochondrial dynamics in a rat model of ischemic stroke. Eur. J. Pharmacol. 861, 172617. 10.1016/j.ejphar.2019.172617 31430457

[B29] Riley R. T. Merrill A. H. Jr. (2019). Ceramide synthase inhibition by fumonisins: A perfect storm of perturbed sphingolipid metabolism, signaling, and disease. J. Lipid Res. 60 (7), 1183–1189. 10.1194/jlr.S093815 31048407 PMC6602133

[B30] Ritchie R. H. Dale Abel E. (2020). Basic mechanisms of diabetic heart disease. Circulation Res. 126, 1501–1525. 10.1161/CIRCRESAHA.120.315913 32437308 PMC7251974

[B31] Russo S. B. Baicu C. F. van Laer A. Geng T. Kasiganesan H. Zile M. R. (2012). Ceramide synthase 5 mediates lipid-induced autophagy and hypertrophy in cardiomyocytes. J. Clin. Investigation 122 (11), 3919–3930. 10.1172/JCI63888 PMC3484448 23023704

[B32] Sanchez A. M. J. Candau R. Bernardi H. (2018). AMP-activated protein kinase stabilizes FOXO3 in primary myotubes. Biochem. Biophysical Res. Commun. 499 (3), 493–498. 10.1016/j.bbrc.2018.03.176 29580989

[B33] Vásquez-Trincado C. García-Carvajal I. Pennanen C. Parra V. Hill J. A. Rothermel B. A. (2016). Mitochondrial dynamics, mitophagy and cardiovascular disease. J. Physiology 594 (3), 509–525. 10.1113/JP271301 PMC5341713 26537557

[B34] Webb A. E. Kundaje A. Brunet A. (2016). Characterization of the direct targets of FOXO transcription factors throughout evolution. Aging Cell 15 (4), 673–685. 10.1111/acel.12479 27061590 PMC4933671

[B35] Weeks K. L. Tham Y. K. Yildiz S. G. Alexander Y. Donner D. G. Kiriazis H. (2021). FoxO1 is required for physiological cardiac hypertrophy induced by exercise but not by constitutively active PI3K. Am. J. Physiology-Heart Circulatory Physiology 320 (4), H1470–H1485. 10.1152/ajpheart.00838.2020 33577435

[B36] World Health Organization (2014). Global status report on noncommunicable diseases 2014. World Health Organization.

[B37] Yoon H. Shaw J. L. Haigis M. C. Greka A. (2021). Lipid metabolism in sickness and in health: Emerging regulators of lipotoxicity. Mol. Cell 81 (18), 3708–3730. 10.1016/j.molcel.2021.08.027 34547235 PMC8620413

[B38] Yun J. Puri R. Yang H. Lizzio M. A. Wu C. Sheng Z.-H. (2014). MUL1 acts in parallel to the PINK1/parkin pathway in regulating mitofusin and compensates for loss of PINK1/parkin. eLife 3, e01958. 10.7554/elife.01958 24898855 PMC4044952

[B39] Zhang B. Huang J. Li H. L. Liu T. Wang Y. Y. Waterman P. (2008). GIDE is a mitochondrial E3 ubiquitin ligase that induces apoptosis and slows growth. Cell Res. 18 (9), 900–910. 10.1038/cr.2008.75 18591963 PMC3156110

[B40] Zhao H. Zhang F. Sun D. Wang X. Zhang X. Zhang J. (2020). Branched-chain amino acids exacerbate obesity-related hepatic glucose and lipid metabolic disorders via attenuating akt2 signaling. Diabetes 69 (6), 1164–1177. 10.2337/db19-0920 32184272

[B41] Zhao Y. Ponnusamy M. Liu C. Tian J. Dong Y. Gao J. (2017). MiR-485-5p modulates mitochondrial fission through targeting mitochondrial anchored protein ligase in cardiac hypertrophy. Biochimica Biophysica Acta - Mol. Basis Dis. 1863 (11), 2871–2881. 10.1016/j.bbadis.2017.07.034 28782654

