# Supplementary figures and images for: Myristate induces mitochondrial fragmentation and cardiomyocyte hypertrophy through mitochondrial E3 ubiquitin ligase MUL1
Source: Front Cell Dev Biol. 2023 Mar 27;11:1072315. doi: 10.3389/fcell.2023.1072315 (PMC10083258; doi:10.3389/fcell.2023.1072315)

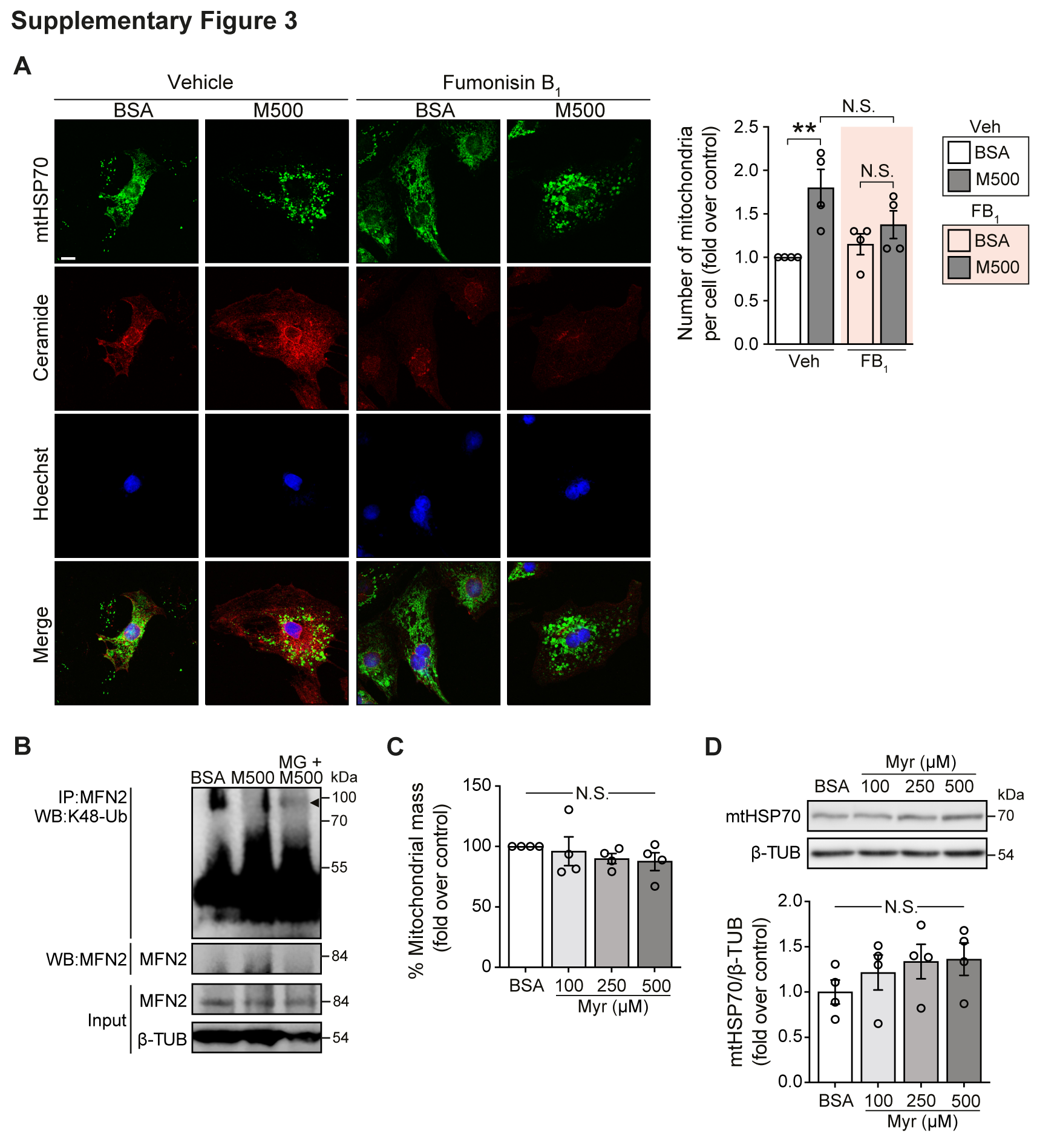

Supplement: Supplementary file 1 [file Image3.TIF]

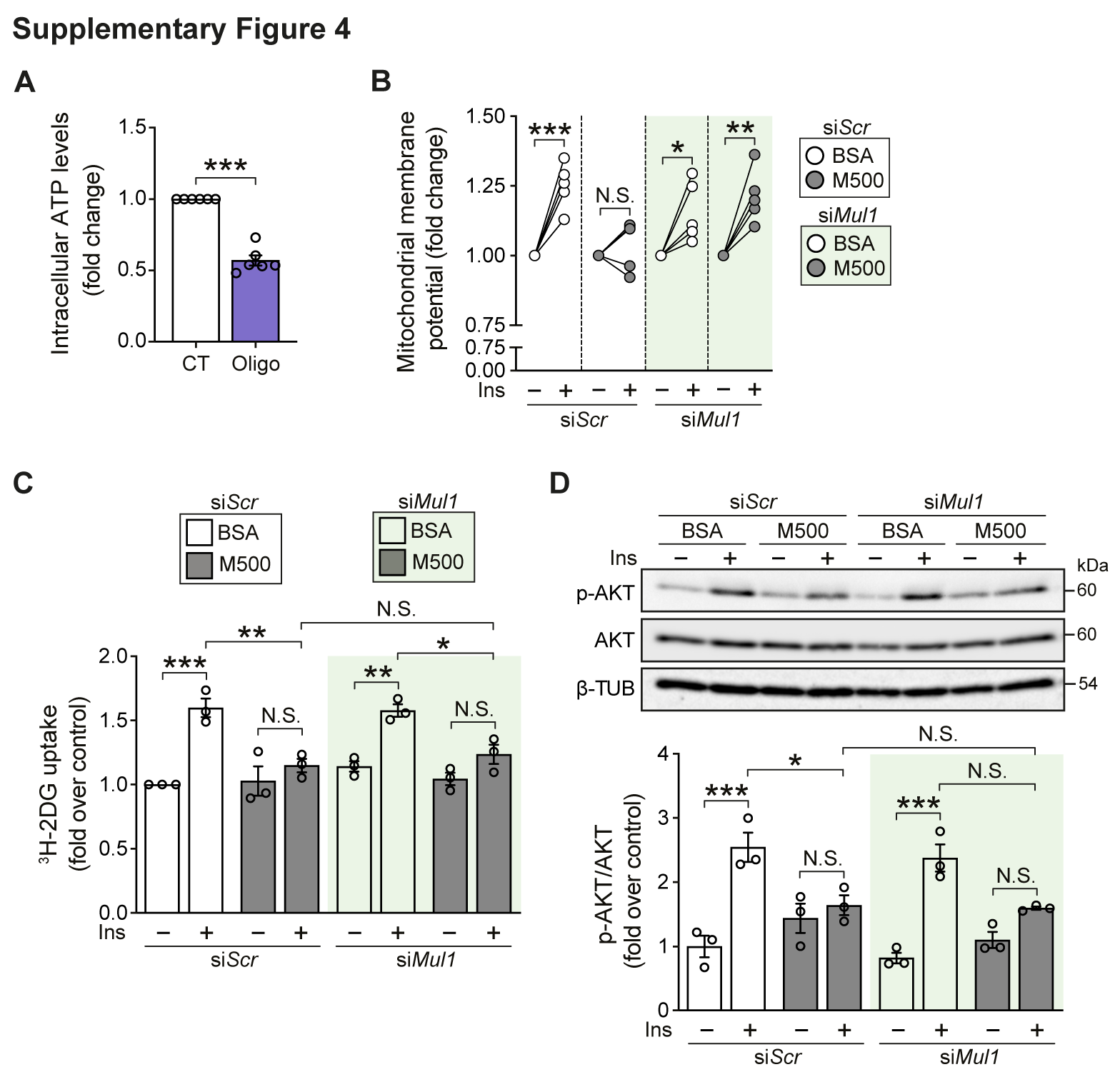

Supplement: Supplementary file 2 [file Image4.TIF]

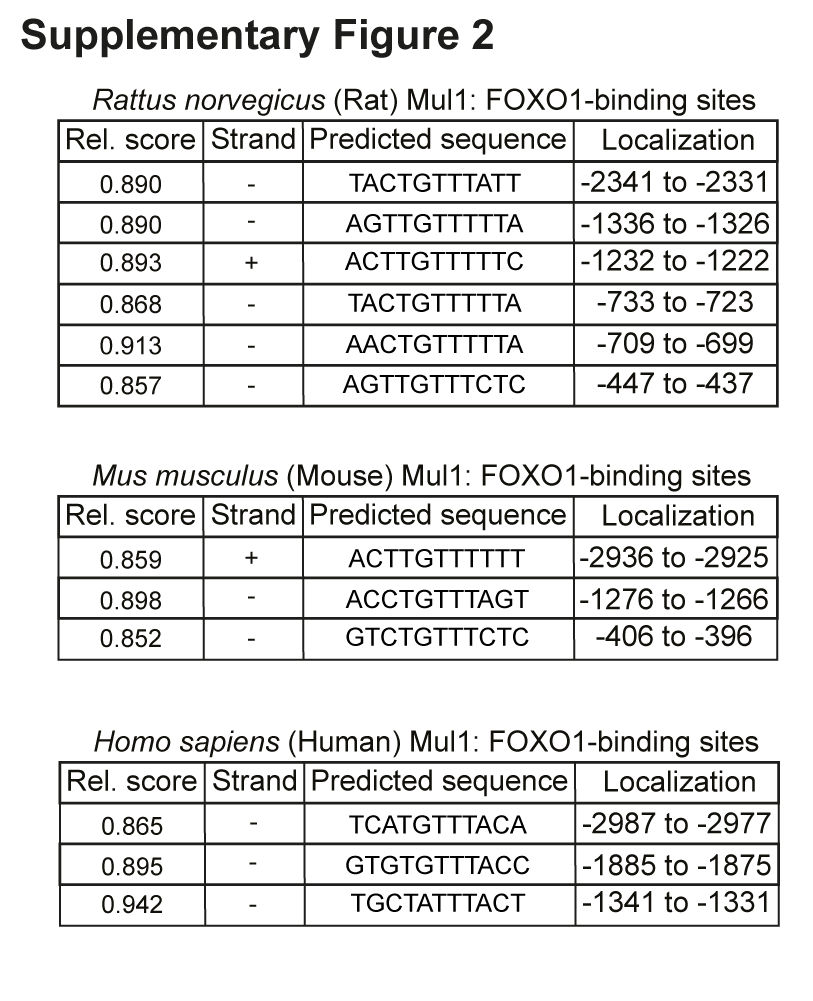

Supplement: Supplementary file 3 [file Image2.TIF]

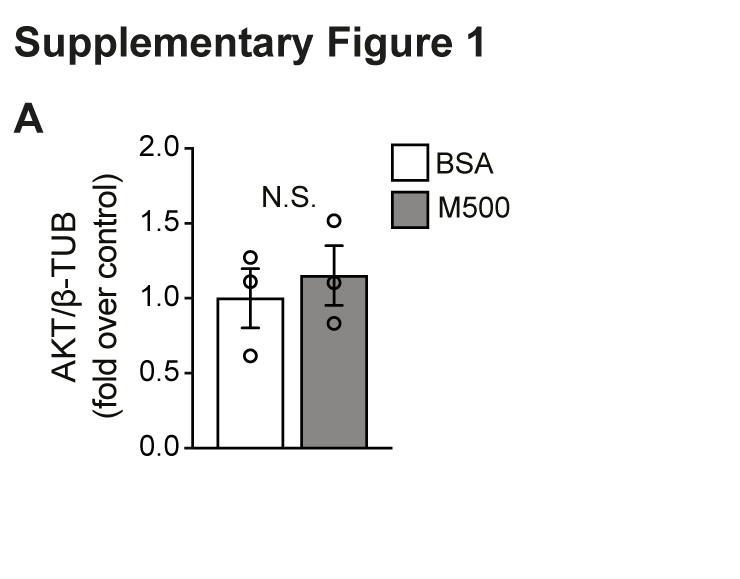

Supplement: Supplementary file 4 [file Image1.TIF]

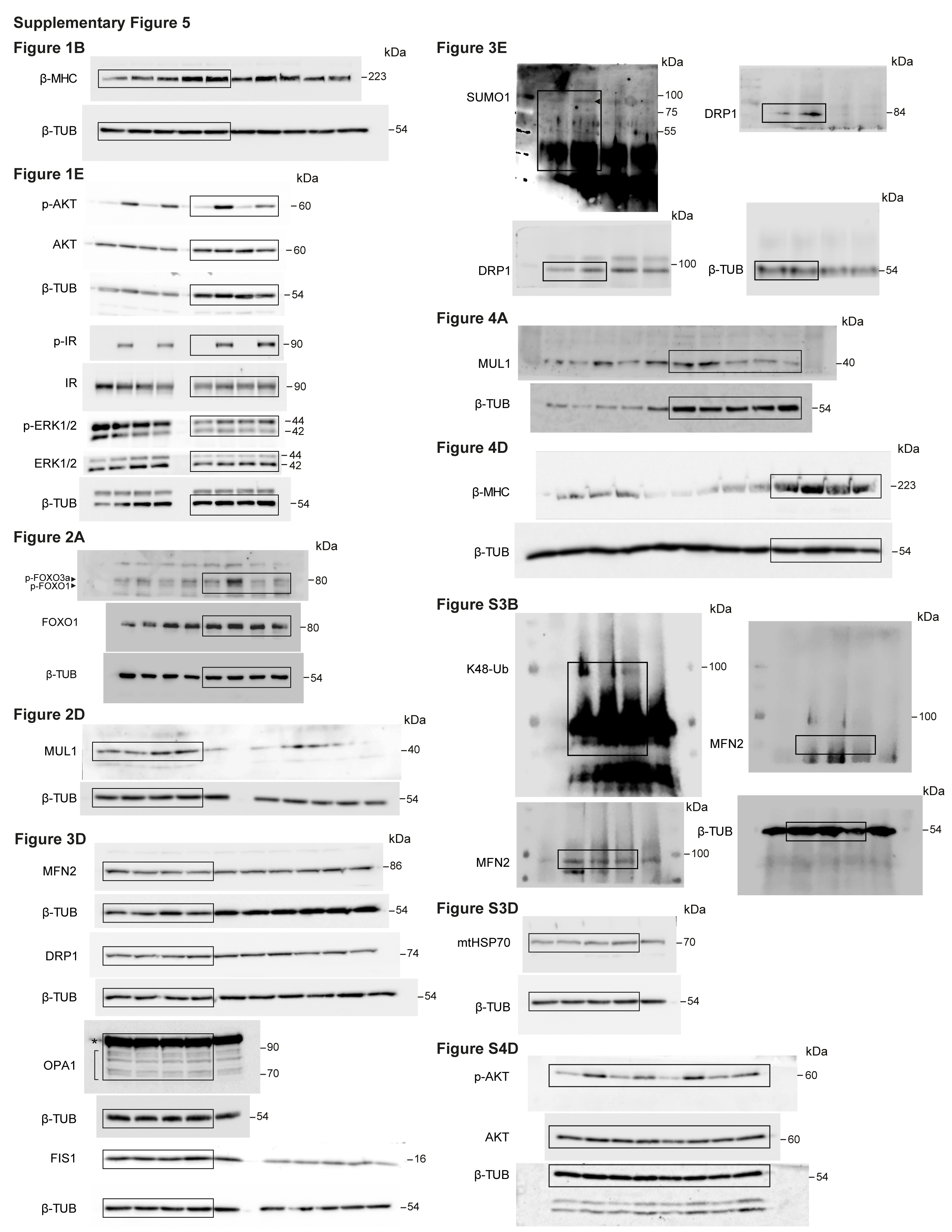

Supplement: Supplementary file 5 [file Image5.TIF]
