# Supplementary material for: Myristate induces mitochondrial fragmentation and cardiomyocyte hypertrophy through mitochondrial E3 ubiquitin ligase MUL1
Source: Front Cell Dev Biol. 2023 Mar 27;11:1072315. doi: 10.3389/fcell.2023.1072315 (PMC10083258; doi:10.3389/fcell.2023.1072315)
Supplement: Supplementary file 6 [file DataSheet1.docx]

Supplementary Material

# Supplementary Figures

## Supplementary Figure 1. Related to Figure 1.

Quantification of AKT/β-TUB (n = 4). β-TUBULIN (β-TUB) was used as a loading control. Individual data points are shown, and bars represent mean ± S.E.M. N.S.: not significant.

## Supplementary Figure 2. Related to Figure 2.

FOXO1 consensus binding sequences found in Mul1 promoter region from rat, mouse and human. Rel. score = Relative score. For more information about this analysis, see subsection 3.14 FOXO1 transcription factor binding sites analysis, in Material and Methods.

## Supplementary Figure 3. Related to Figure 3.

(A) *Left*: Representative immunofluorescence images of NRVM treated with BSA or myristate 500 μM (M500) for 24 h, using mtHSP70 antibody (green) to evaluate mitochondrial network, ceramide antibody (red) and the Hoechst nuclear stain (blue). Scale bar: 10 μM. *Right*: Quantification of mitochondrial number (n = 4). (B) MFN2 ubiquitination (K48 specific linkage) assessed by immunoblot after immunoprecipitation of MFN2 protein from NRVM treated with BSA, myristate 500 μM (M500) for 24 h and M500 for 24 h plus MG132 (MG) for 1 h. A representative image is shown from three independent experiments with similar outcomes. (C) Flow cytometry analysis of mitochondrial mass (using MitoTracker Green) of cardiomyocytes were treated with BSA 100 μM or Myristate (Myr) 100, 250 and 500 μM, for 24 h (n = 4). (B) *Top*: Representative immunoblot of mtHSP70, detected in cardiomyocytes treated with Myr 100, 250 and 500 μM, for 24 h. β-TUBULIN (β-TUB) was used as a loading control. *Bottom*: Quantification of mtHSP70/β‑TUB (n = 4). In all panels: individual data points are shown, and bars represent mean ± S.E.M. N.S.: not significant.

## Supplementary Figure 4. Related to Figure 4.

Measurement of intracellular ATP levels of cardiomyocytes untreated or treated with 1μM Oligomycin (n = 5). (B) Flow cytometry analysis of mitochondrial membrane potential (using TMRM) of cardiomyocytes treated with siRNA Scramble (siScr) or siRNA for *Mul1* (si*Mul1*), incubated with BSA or myristate 500 μM (M500) for 24 h and stimulated with insulin (Ins) 10 nM for 3 h or non-stimulated (n = 5). (C) ^3^H-2-deoxyglucose (DG) uptake of cardiomyocytes treated with siScr or si*Mul1*, incubated with BSA or M500 for 24 h and stimulated with insulin (Ins) 10 nM for 15 min or non-stimulated (n = 3). (D) *To*p: Representative immunoblots of p-AKT (Ser473) and total AKT from cardiomyocytes treated with BSA or myristate 500 μM (M500) for 24 h, unstimulated or stimulated with insulin 10 nM for 15 min. β-TUBULIN (β-TUB) was used as a loading control. *Bottom*: Quantification of p-AKT/AKT (n = 3). In all panels: individual data points are shown, and bars represent mean ± S.E.M. Statistical comparison: *p<0.05, **p<0.01 and ***p<0.01. N.S.: not significant.

## Supplementary Figure 5

Uncropped images of representative immunoblots used in this article.
